# Perinatal Antibiotic Exposure Affects the Transmission between Maternal and Neonatal Microbiota and Is Associated with Early-Onset Sepsis

**DOI:** 10.1128/mSphere.00984-19

**Published:** 2020-02-19

**Authors:** Ping Zhou, Yanxia Zhou, Bin Liu, Zhenchao Jin, Xueling Zhuang, Wenkui Dai, Zhenyu Yang, Xin Feng, Qian Zhou, Yanhong Liu, Ximing Xu, Lian Zhang

**Affiliations:** a Department of Neonatology, Jinan University-affiliated Shenzhen Baoan Women's and Children's Hospital, Shenzhen, China; b WeHealthGene Institute, Shenzhen, China; c School of Statistics and Data Science, NanKai University, Tianjin, China; d Key Laboratory for Medical Data Analysis and Statistical Research of Tianjin, Tianjin, China; e Research Laboratory, Jinan University-affiliated Shenzhen Baoan Women's and Children's Hospital, Shenzhen, China; Antimicrobial Development Specialists, LLC

**Keywords:** perinatal antibiotic exposure, maternal vaginal microbiota, meconium microbiota, early-onset sepsis, perinatal antibiotic treatment

## Abstract

Intrapartum antibiotic prophylaxis reduces the risk of infection to a mother and neonate, but antibiotic-mediated maternal and neonatal microbiota dysbiosis increases other health risks to newborn infants. We studied the impact of perinatal antibiotic prophylaxis on the microbiota in mothers and newborns with full-term or preterm delivery. Ninety-eight pregnant women and their neonates were divided into the following four groups: full term without antibiotic exposure (FT), full term with antibiotic exposure (FTA), preterm without antibiotic exposure (PT), and preterm with antibiotic exposure (PTA). Bacterial composition was analyzed by sequencing the 16S rRNA gene from maternal vaginal swabs (V) and neonatal meconium (F). The results showed that in maternal vaginal and neonatal meconium microbiota, FT and PT groups had a higher load of Lactobacillus spp. than did the FTA and PTA groups. In addition, whether in the mother or newborn, the dissimilarity in microbiota between FT and PT was the lowest compared to that between other groups. Compared to the FT and PT groups, the dissimilarity in microbial structures between the vagina and meconium decreased in the FTA and PTA groups. The health outcome of infants reveals an association between early-onset sepsis and antibiotic-mediated microbiota dysbiosis. In conclusion, perinatal antibiotic exposure is related to the establishment of gut microbiota and health risks in newborns. Promoting the rational usage of antibiotics with pregnant women will improve neonatal health.

**IMPORTANCE** Perinatal antibiotic prophylaxis is an effective method for preventing group B *Streptococcus* (GBS) infection in newborns. Antibiotic exposure unbalances women’s vaginal microbiota, which is associated with the establishment of the newborn gut microbiota. However, the influence of perinatal antibiotic exposure on neonatal gut microbiota colonization and health outcomes remains unclear. In this study, we found that perinatal antibiotic exposure induced microbiota dysbiosis in a woman’s vagina and the neonatal gut, and we highlight a significant decrease in the abundance of *Lactobacillus* spp. The influence of antibiotic use on the microbiota was greater than that from gestational age. Additionally, full-term newborns without antibiotic exposure had no evidence of early-onset sepsis, whereas in full-term or preterm newborns with antibiotic exposure before birth, at least one infant was diagnosed with early-onset sepsis. These results suggest an association between perinatal antibiotic exposure and microbial dysbiosis in maternal vaginal and neonatal gut environments, which may be related to the occurrence of early-onset sepsis.

## Abstract (precis)

Perinatal antibiotic prophylaxis is an effective method for preventing group B *Streptococcus* (GBS) infection in newborns. Antibiotic exposure unbalances women’s vaginal microbiota, which is associated with the establishment of the newborn gut microbiota. However, the influence of perinatal antibiotic exposure on neonatal gut microbiota colonization and health outcomes remains unclear. In this study, we found that perinatal antibiotic exposure induced microbiota dysbiosis in a woman’s vagina and the neonatal gut, and we highlight a significant decrease in the abundance of *Lactobacillus* spp. The influence of antibiotic use on the microbiota was greater than that from gestational age. Additionally, full-term newborns without antibiotic exposure had no evidence of early-onset sepsis, whereas in full-term or preterm newborns with antibiotic exposure before birth, at least one infant was diagnosed with early-onset sepsis. These results suggest an association between perinatal antibiotic exposure and microbial dysbiosis in maternal vaginal and neonatal gut environments, which may be related to the occurrence of early-onset sepsis.

## OBSERVATION

Multiple studies demonstrate that the transmission of a mother’s microbiota to infants differs with delivery mode and antibiotic exposure (1, 2). Antibiotics have significant effects on the microbiota in different body sites, such as the gut and vagina (3). In addition, the administration of antibiotics causes antibiotic-resistant bacteria (4) and microbiota disequilibrium in the vaginal tract and neonate gut (5), which increase the risk of neonatal diseases (6). Intrapartum antibiotic prophylaxis is widely applied as a prevention strategy against group B Streptococcus (GBS) infection in China (7). In this study, we aim to assess how perinatal antibiotic exposure impacts the microbiota transmission between the mother and infant, as well as the relationship between the microbiota and risk of early-onset sepsis (EOS) in neonates.

## 

### Data description.

On average, we generated 43,951 ± 8,572 tags and 357 ± 228 operational taxonomic units (OTUs) for vaginal swabs, as well as 41,461 ± 11,192 tags and 319 ± 192 OTUs for meconium samples. Additionally, no nucleic acids were amplified from the negative controls. Permutational multivariate analysis of variance (PERMANOVA) showed that the time of first maternal antibiotic exposure did not affect the vaginal and neonatal microbiota composition in each group (*P* > 0.050).

### *Lactobacillus* load in vaginal microbiota was related to perinatal antibiotic exposure.

**FIG 1:**
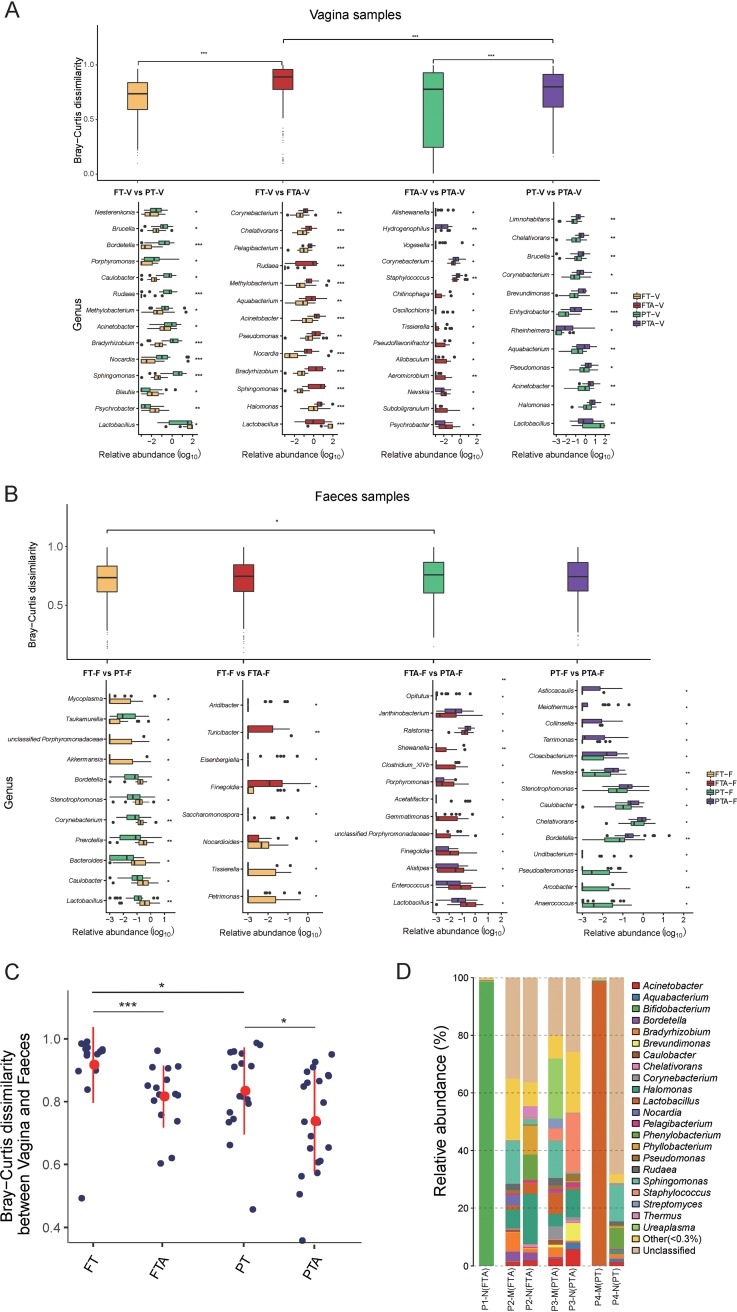
Bray-Curtis dissimilarity and microbiota composition among four groups in vaginal and meconium microbiota. (A) In vaginal microbiota, Bray-Curtis dissimilarity (top) and the distinct genus (bottom, *P < *0.05) between groups after pairwise comparison. (B) In meconium microbiota, the Bray-Curtis dissimilarity (top) and the distinct genus (bottom, *P < *0.05) between groups after pairwise comparison. (C) Bray-Curtis dissimilarity of microbiota between vagina and meconium in four groups. (D) Neonatal meconium (N) and maternal vaginal (M) microbiota composition of EOS infants and their mothers (*, *P < *0.050; **, *P < *0.010; and ***, *P *< 0.001).

The dissimilarity in the vaginal microbiota between FT-V and FTA-V was significantly higher than that between FT-V and PT-V (*P* = 0.001) and between PT-V and PTA-V (*P* = 0.000) (Fig. 1A, top). For vaginal microbiota composition, there were significant differences in *Lactobacillus* load among four groups. Both for pregnant women with full-term and preterm delivery, antibiotic exposure caused a dramatic reduction in the load of *Lactobacillus* spp. in the vaginal microbiota (Fig. 1A, bottom; FT-V, 66.17% ± 37.43% versus FTA-V, 12.86% ± 23.12%, *P* = 0.000; PT-V, 35.31% ± 38.65% versus PTA-V, 11.02% ± 21.40%, *P* = 0.036). In addition, for vaginal swabs without antibiotic exposure, *Lactobacillus* spp. had about a 1.8-fold higher abundance in full-term (FT-V, 66.17% ± 37.43%) than in preterm (PT-V, 35.31% ± 38.65%, *P = *0.010) infants (Fig. 1A, bottom).

### Gestational age was associated with *Lactobacillus* load in neonate meconium.

For meconium microbial samples, the dissimilarity between FTA-F and PTA-F was slightly higher than that between the FT-F and PT-F groups (Fig. 1B, top) (*P* = 0.026). Regardless of antibiotic exposure before delivery, the *Lactobacillus* load in meconium microbiota with full-term delivery was significantly higher than that with preterm delivery (Fig. 1B, bottom; FT-F, 0.74% ± 1.07% versus PT-F, 0.35% ± 0.74%, *P* = 0.007; FTA-F, 0.76% ± 1.18% versus PTA-F, 0.19% ± 0.35%, *P = *0.007). Nevertheless, there were no significant differences in *Lactobacillus* load between FT-F and FTA-F (Fig. 1B, bottom; FT-F, 0.74% ± 1.07% versus FTA-F, 0.76% ± 1.18%, *P = *0.423) or between PT-F and PTA-F (Fig. 1B, bottom; PT-F, 0.35% ± 0.74% versus PTA-F, 0.19% ± 0.35%, *P = *0.237).

### The dissimilarity of microbiota between the vagina and meconium was affected by perinatal antibiotic exposure and gestational age.

For paired mother and newborn groups with or without antibiotic exposure, the dissimilarity between vaginal and meconium microbial samples was higher with full-term delivery than with preterm delivery (Fig. 1C). When delivered at the same gestational age, full-term or preterm, the dissimilarity between vaginal and meconium samples was higher in paired mother and newborn groups without antibiotic exposure before delivery (Fig. 1C).

### Imbalanced microbiota in EOS neonates’ meconium and their maternal vagina.

Among the mothers of four neonates who were diagnosed with EOS, three mothers (P1-M to P3-M) experienced antibiotic exposure because of a high risk of group B *Streptococcus* (GBS) infection. For the mother of neonate P1 (P1-M), cefazolin was administered 11 h before full-term delivery, and Bifidobacterium spp. (98.55%) were dominant in the meconium microbiota (Fig. 1D, P1-N [FTA]). Nevertheless, the lack of vaginal microbiota information from neonate P1’s mother was due to a failure in sample amplification. The mother of neonate P2 was treated by cefazolin for 3 h before full-term delivery, and the vaginal microbiota showed a low load of *Lactobacillus* spp. (1.36%) (Fig. 1D, P2-M [FTA]). In addition, a high level of Halomonas spp. (17.37%) was identified in neonate P2’s meconium microbiota (Fig. 1D, P2-N [FTA]). The mother of neonate P3 was administered with cefazolin in 20 h before preterm delivery, and her vaginal microbiota (Fig. 1D, P3-M [PTA]) was characterized by a low load of *Lactobacillus* spp. (7.33%) and a high level of Staphylococcus spp. (4.27%) compared to the vaginal microbiota of mothers without antibiotic exposure in the PT-V group. In the meconium microbiota of neonate P3, *Staphylococcus* (20.66%) was the predominant genus (Fig. 1D, P3-N [PTA]). For neonate P4, the mother received no antibiotic exposure before preterm delivery, and the dominant genus in the vaginal microbiota was *Lactobacillus* (98.46%) (Fig. 1D, P4-M [PT]); the meconium of neonate P4 was populated by Sphingomonas spp. (12.48%) and by 68.19% of OTUs which could not be classified to known taxonomic units (Fig. 1D, P4-N [PT]). A cultivation experiment for blood samples from the above-mentioned four neonates showed that neonate P4 was positive only for Escherichia coli, and blood cultivations of the other three EOS neonates were negative.

Gestational age and perinatal antibiotic exposure impact the vaginal microbiota (2, 8). Several studies demonstrate that the decreased load and diversity of *Lactobacillus* spp. in vaginal microbiota are associated with a high risk of preterm delivery and impose negative effects on mother-to-infant microbiota transmission (1, 9). Consistent with prior studies (9, 10), we identified a significantly reduced load of vaginal *Lactobacillus* spp. in mothers with preterm delivery or following antibiotic exposure (11, 12). Given the robust effect of antibiotic exposure on the microbiota (13), our further analysis showed that antibiotic exposure seems to impose a stronger effect on vaginal microbiota than does gestational age.

A series of studies emphasize the contribution of mother-to-infant microbiota transmission to health development (14). Our study indicates that meconium microbiota in preterm delivery is characterized by higher levels of facultative anaerobic microorganisms and reduced levels of strict anaerobes such as *Bifidobacterium*, Bacteroides, and Atopobium spp. compared to full-term delivery (2). Nevertheless, unlike with the vaginal microbiota, perinatal antibiotic exposure has no obvious effect on the *Lactobacillus* load in meconium. This may be partially explained by individual differences as well as selective colonization of vagina-derived microbial components in the gut of neonates (15).

In line with previous studies, our research indicates that newborns at full term without antibiotic exposure bear a lower health risk for conditions such as for EOS or respiratory distress than do preterm or antibiotic-exposed neonates (16, 17). Gut microbiota dysbiosis is a high risk factor for EOS, as a prior study showed (18). In our study, distinct bacterial components were identified for each EOS patient, such as a high load of *Bifidobacterium* and *Staphylococcus* spp. in the gut microbiota as well as individual-specific vaginal microbiota. This preliminary result provides additional insights into the necessity of assessing individual-specific microbiota transmission when considering microbiota as an intervention target to prevent EOS. However, a further explanation for the impact of perinatal antibiotic exposure and the etiology of EOS is limited by insufficient sample size for each group.

In conclusion, perinatal antibiotic exposure may be associated with vaginal microbiota dysbiosis, highlighted by substantially reduced levels of *Lactobacillus* spp., which may elevate the risk for vaginal GBS infection and preterm delivery. In addition, full-term gestational age and absence of perinatal antibiotic exposure seem to be protective factors in the transmission of dysbiosis gut microbiota and EOS.

This study was approved by the Shenzhen City Baoan District Women and Children’s Hospital Ethics Committee with the registration number LLSC 2018-12-8. All pregnant women provided informed consent when conducting sampling and follow-up investigation.

The pregnant women were recruited according to the following criteria: (i) 20 to 35 years of age and no history of smoking, taking drugs, or alcoholic intemperance; (ii) normal rate of weight gain, body mass index, hepatorenal function, myocardial enzymes, body glucose, serum total cholesterol, and triglyceride levels during pregnancy; (iii) no family allergy history, abnormal complications, or chronic diseases in pregnancy (including diabetes, digestive system disease, chronic infectious disease, and urine and reproductive diseases); (iv) absence of antibiotic exposure before our study; and (v) a vaginal delivery. The paired neonates were excluded as per the following criteria: (i) had birth asphyxia or were diagnosed with hypoxic-ischemic encephalopathy/intracranial bleeding; (ii) had a genetic metabolic disease or congenital malformation; (iii) an antibiotic or microecological preparation was used before sampling; (iv) were a twin or multiple birth; or (v) had noninfectious disease, such as neonatal cholestasis, neonatal hepatitis, and hemolytic diseases of newborns. Before birth within 48 h, empirical cefazolin was used through intravenous injection with 2 g every 12 h on pregnant women for the prevention of group B *Streptococcus* (GBS) newborn infection.

In total, 98 pregnant women and their neonates were included in our study and stratified into four groups, as follows: full term (gestational age, ≥37 weeks) without antibiotic exposure (FT, *n* = 23), full term with antibiotic exposure (FTA, *n* = 27), preterm (gestational age, <37 weeks) without antibiotic exposure (PT, *n* = 23), and preterm with antibiotic exposure (PTA, *n* = 25). One month after birth, a follow-up investigation was conducted to assess the risk of EOS (Table 1), which was diagnosed according to a clinical guide (19, 20).

**TABLE 1:** Clinical information for the four patient groups

Characteristic^*a*^	Data for group:			
	FT	FTA	PT	PTA
No. of vaginal samples	18	18	22	24
No. of meconium samples	21	26	21	24
No. of maternal GBS infections	0	5	0	2
No. of premature rupture of membranes	0	21	7	20
No. with amniotic fluid contamination (grade III)	3	8	4	0
Gestational age (mean ± SD) (wk)	39.42 ± 0.91	39.22 ± 1.03	35.17 ± 1.31	34.96 ± 1.51
Neonatal sex (no. of males: no. of females)	16:8	15:12	13:11	11:14
Neonatal wt (mean ± SD) (g)	3,106.25 ± 348.72	3,368.52 ± 344.93	2,558.33 ± 405.03	2,471.60 ± 329.27
Neonatal WBC count (mean ± SD) (10^9^/liter)	20.10 ± 5.51	18.20 ± 7.19	13.75 ± 6.85	12.57 ± 5.39
Neonatal CRP (mean ± SD) (mg/liter)	1.23 ± 1.26	3.69 ± 8.98	0.99 ± 1.82	2.284 ± 6.34
No. with low birth wt	1	0	9	12
No. with early-onset sepsis	0	2	1	1

a GBS, group B *Streptococcus*; WBC, white blood cell; CRP, C-reactive protein.

Vaginal samples were collected from the posterior fornix under direct visualization using three swabs before delivery and immediately stored at –80°C without buffer (Kang Jian, Jiangsu, China). The collected meconium (obtained from a sterile single-use diaper) was stored at –80°C without buffer in 30 min (iClean HCY, Shenzhen, China). Enveloped sampling materials were also collected as negative controls for the assessment of DNA contamination. The microbial genomic DNA was extracted from vaginal and meconium samples using the DNeasy PowerSoil kit (Qiagen, Hilden, Germany), according to the manufacturer’s protocol. A Qubit fluorometer (Thermo Fisher Scientific, Singapore) was used to qualify isolated DNA. The V4 region of the 16S rRNA gene was amplified utilizing the 515F/806R primers and sequenced on a MiSeq platform (Illumina, San Diego, CA, USA), with a 2 × 250-bp cartridge. Raw sequencing reads were filtered and clustered into operational taxonomic units (OTUs) with 97% similarity via USEARCH (21) and then classified taxonomically by alignment with the Ribosomal Database Project database (22). Bray-Curtis dissimilarity between different samples and nonparametric permutational multivariate analysis of variance (PERMANOVA) for assessing antibiotic use time on microbiota composition (false-discovery rate [FDR], <0.05) were calculated using the vegan version 2.5-3 package in R 3.5.1. A Wilcoxon rank-sum test was used for comparative analysis between groups.

### Data availability.

The raw reads have been deposited to GenBank under BioProject number PRJNA553858.
